# Evaluation of Anti-Inflammatory Properties of Isoorientin Isolated from Tubers of* Pueraria tuberosa*

**DOI:** 10.1155/2017/5498054

**Published:** 2017-01-24

**Authors:** Kotha Anilkumar, Gorla V. Reddy, Rajaram Azad, Nagendra Sastry Yarla, Gangappa Dharmapuri, Anand Srivastava, Mohammad A. Kamal, Reddanna Pallu

**Affiliations:** ^1^School of Life Sciences, University of Hyderabad, Hyderabad 500046, India; ^2^National Institute of Animal Biotechnology, Hyderabad 500049, India; ^3^Department of Biochemistry and Bioinformatics, School of Life Sciences, Institute of Science, GITAM University, Visakhapatnam, Andhra Pradesh 530 045, India; ^4^King Fahd Medical Research Center, King Abdulaziz University, P.O. Box 80216, Jeddah 21589, Saudi Arabia; ^5^Enzymoics, 7 Peterlee Place, Hebersham, NSW 2770, Australia; ^6^Novel Global Community Educational Foundation, Hebersham, NSW, Australia

## Abstract

Inflammation is the major causative factor of different diseases such as cardiovascular disease, diabetes, obesity, osteoporosis, rheumatoid arthritis, inflammatory bowel disease, and cancer. Anti-inflammatory drugs are often the first step of treatment in many of these diseases. The present study is aimed at evaluating the anti-inflammatory properties of isoorientin, a selective cyclooxygenase-2 (COX-2) inhibitor isolated from the tubers of* Pueraria tuberosa*, in vitro on mouse macrophage cell line (RAW 264.7) and in vivo on mouse paw edema and air pouch models of inflammation. Isoorientin reduced inflammation in RAW 264.7 cell line in vitro and carrageenan induced inflammatory animal model systems in vivo. Cellular infiltration into pouch tissue was reduced in isoorientin treated mice compared to carrageenan treated mice. Isoorientin treated RAW 264.7 cells and animals showed reduced expression of inflammatory proteins like COX-2, tumor necrosis factor-*α* (TNF-*α*), interleukin-6 (IL-6), 5-lipoxygenase (5-LOX), and interleukin 1-*β* (IL-1-*β*) both in vitro and in vivo. The antioxidant enzyme levels of catalase and GST were markedly increased in isoorientin treated mice compared to carrageenan treated mice. These results suggest that isoorientin, a selective inhibitor of COX-2, not only exerts anti-inflammatory effects in LPS induced RAW cells and carrageenan induced inflammatory model systems but also exhibits potent antioxidant properties.

## 1. Introduction

Inflammation is a cellular, immune, and metabolic response to injury/infection. It is a normal protective vascular connective tissue response to eliminate the cause of injury and clean up the dead and dying cells but when it occurs in uncontrolled or inappropriate manner it results in pathogenesis of several disorders which include cardiovascular, respiratory, neurological, and many lifestyle diseases. Inflammation is a complex interplay of cellular and particulate mediators, which include chemokines, plasma enzymes, lipids, and cytokines. Among these the lipid mediators such as eicosanoids, the oxygenated metabolites of arachidonic acid formed via the cyclooxygenase (COX) and lipoxygenase (LOX) pathways, play a predominant role in mediating the inflammatory disorders. The anti-inflammatory drugs are being targeted against COX pathway. These include the conventional nonsteroidal anti-inflammatory drugs (NSAIDs) that target both COX-1 and COX-2 and the selective COX-2 inhibitors (COXIBs). Although these drugs are effective in controlling signs of inflammation, number of adverse effects encountered is the biggest limitation to their use [1, 2]. These include the gastric side effects of traditional NSAIDs and the cardiac side effects of COXIBs.

Traditional medicinal practices have been known for millennia for the treatment of various ailments [3, 4]. Over three-quarters of world population are relying mainly on plants and plant extracts for health care [3–5]. Medicinal plants produce bioactive compounds used mainly for medicinal purposes. Recently it was shown that tuberostemonine N, isolated from* Stemona tuberosa*, suppresses cigarette smoke induced subacute lung inflammation in mice [6]. Similarly it was shown that natural products like (7R, 8S)-9-acetyl-dehydrodiconiferyl alcohol (ADDA), isolated from* Clematis armandii* stems [7] and mitraphylline from* Uncaria tomentosa* [8], exert anti-inflammatory properties. The present study is aimed at determining the anti-inflammatory effects of isoorientin isolated from the tubers of* Pueraria tuberosa*.* P. tuberosa* herb is used as a cardiotonic, galactagogue, and diuretic, as well as for fertility control in folk medicine. The tuber extracts are known to have antioxidant, hypoglycemic, hypolipidemic, anti-inflammatory, and in vivo immunomodulatory activities [9–12]. Earlier, we have shown that isoorientin selectively inhibits COX-2 suggesting its potential as a promising anti-inflammatory drug candidate [13]. Present study evaluates the anti-inflammatory potential of isoorientin in vitro on mouse macrophage cell line, RAW 264.7, and on paw edema and air pouch models of inflammation in vivo.

## 2. Materials and Methods

MTT (3-(4,5-dimethylthiazol-2-yl)-2,5-diphenyltetrazolium bromide), trypsin-EDTA, Tris, ethylenediaminetetraacetic acid (EDTA), diethyldithiocarbamate (DDC), Tween-20, hematin, glycerol, phenol, and ammonium sulphate were purchased from Sigma Chemical Co. (St. Louis, MO). Celecoxib was a generous gift from Unichem Laboratories (Mumbai, India). Carrageenan was purchased from Sigma-Aldrich (St. Louis, USA). Polyclonal antibodies to COX-2, IL-6, 5-LOX, IL-1*β*, and TNF-*α* were purchased from Santa Cruz Biotechnology (California, USA). iNOS antibody was from Thermo Fisher Scientific Inc. All other chemicals and solvents were of analytical grade and purchased from authorized standard companies. Isoorientin was isolated from the methanolic extracts of tubers of* P. tuberosa* [13].

### 2.1. Cell Culture

RAW 264.7 (murine macrophages) cell line was obtained from National Centre for Cell Science (NCCS), Pune. The cells were maintained in a humidified atmosphere with 5% CO_2_ at 37°C. Medium for all the cell lines was DMEM supplemented with 10% heat inactivated fetal bovine serum (FBS), 100 IU/mL penicillin, 100 *μ*g/mL streptomycin, and 2 mM L-glutamine.

### 2.2. Effect of Isoorientin on Cell Viability

RAW 264.7 cells viability in the presence and absence of isoorientin was assessed using MTT assay. Cells were treated with different concentrations of isoorientin (25 nM to 100 *μ*M) for 16 h and then the cell viability was assessed by the mitochondrial-dependent reduction of 3-(4,5-dimethylthiazol-2-yl)-2,5-diphenyltetrazolium bromide (MTT) (0.5%) to purple formazan. Cells were then incubated with MTT (0.5%) for 4 h at 37°C. The medium was then removed by aspiration and formazan crystals were dissolved in DMSO. The extent of the reduction of MTT was quantified by measurement of absorbance at 570 nm using microtiter plate reader.

### 2.3. SDS-PAGE and Western Blotting

An equal quantity of cytosolic/nuclear proteins from each treatment (75 *μ*g of total protein/lane) was resolved on 8–12% SDS-PAGE gels and then transferred onto nitrocellulose membranes. Membranes were stained with 0.5% Ponceau in 1% acetic acid to confirm equal loading. The membranes were blocked with 5% w/v nonfat dry milk and then incubated with the primary antibodies in 10 mL of antibody-diluted buffer (Tris-buffered saline and 0.05% Tween-20 with 5% milk) with gentle shaking at 4°C for 8–12 h and then incubated with respective conjugated secondary antibodies. Signals were detected using Western blot detection reagents.

### 2.4. Preparation of Cytoplasmic and Nuclear Extracts

RAW 264.7 cells were cultured in 6-well plates (4 × 10^6^ cells/well) with or without LPS (1 *μ*g/mL) and in the presence or absence of isoorientin (0–25 *μ*M). The cytoplasmic and nuclear protein extracts were prepared for measuring the protein levels by Western blotting and enzyme immunoassay (EIA). Briefly, after culture the cells were collected and washed twice with cold PBS and lysed in 400 *μ*L of cold buffer A (HEPES 10 mmol/L pH 7.9, KCl 10 mmol/L, 1 mM EDTA, phenylmethanesulphonylfluoride (PMSF) 1 mmol/L, 1 mM EGTA, dithiothreitol (DTT) 1 mmol/L, aprotinin 1 mg/L, leupeptin 1 mg/L, and pepstatin A 1 mg/L). After 15 min incubation on ice, 0.1% NP-40 was added to the homogenates and the tubes were vigorously rocked for 1 min. Then the homogenates were centrifuged (20,800 ×g, 5 min) in a microcentrifuge at 4°C. The supernatant fluid (cytoplasmic extracts) was collected and stored in aliquots at −70°C. The nuclear pellets were washed once with cold buffer A, then suspended in 50 *μ*L of cold buffer B (HEPES 20 mmol/L, pH 7.9, NaCl 420 mmol/L, edetic acid 0.1 mmol/L, egtazic acid 0.1 mmol/L, PMSF 1 mmol/L, DTT 1 mmol/L, aprotinin 1 mg/L, leupeptin 1 mg/L, and pepstatin A 1 mg/L), and vigorously rocked at maximum speed at 4°C for 30 min. The solution was clarified by centrifugation at 20,800 ×g for 5 min, and the supernatant fluid (nuclear extract) was stored in aliquots at −70°C. The protein concentration was determined according to the Bradford method [14].

### 2.5. Experimental Animals

Adult Balb/c male mice weighing 20–25 g were used for all the experiments in the present study. They were fed with a standard chow pellet diet, had free access to water, and were maintained on a 12 : 12-h light-dark cycles. All procedures in this study were approved by the Animal Ethical Committee of the National Institute of Animal Biotechnology.

### 2.6. Paw Edema Model

The study was performed on carrageenan induced paw edema in Balb/c mice with swelling measured using electronic vernier calipers. Paw edema was induced in Balb/c mice by subcutaneous injection of carrageenan (0.1 mL of 1% solution w/v in 0.9% saline) into subplantar region of the left hind paw and these animals were divided into 5 different groups (6 mice in each group) as described below in administration of isoorientin. Edema was calculated as the average difference of paw thickness (mm) in treated groups compared with that in control groups (DMSO treated).

### 2.7. Air Pouch Model of Inflammation

Carrageenan treated mice air pouch model of inflammation was developed as described previously [15]. Air cavities were produced by subcutaneous injections of 5 mL of sterile air into the intracapsular area on the dorsal side of the animal. An additional 3 mL of air was injected into the cavity every three days. Seven days after the initial air injection, 0.5 mL of 1.5% (w/v) solution of carrageenan dissolved in saline was injected directly into the pouch to produce an inflammatory response. For the time course studies, animals were sacrificed by cervical dislocation at various time points after the injection. Pouch tissue was carefully dissected and cut open to aspirate the inflammatory exudates into graduated tubes. The pouch lining was separated from the muscle and dissected out and rinsed in saline before processing further. Cell population in the pouch cavity was measured by gavage of about 2 mL of saline into the pouch repeatedly. This procedure ensures the complete recovery of cells from the pouch. For cell counting the collected fluid was centrifuged and the cell pellet was washed in RPMI medium twice to remove the debris and dissolved in saline and then counted on hemocytometer.

### 2.8. Administration of Isoorientin

In the case of paw edema model the isoorientin or celecoxib was given intraperitoneally and carrageenan was injected into the paw directly one hour later. In air pouch model all the treatments were given along with carrageenan directly into the pouch cavity. Isoorientin was injected three hours earlier than injection of carrageenan into the pouch cavity. Isoorientin and celecoxib were administered into the mice air pouch. The stock solutions of isoorientin (100 mg/mL) and celecoxib (100 mg/mL) were prepared in DMSO and further dilutions were made at the time of treatments. Animals were divided, into 5 different groups as follows: control (DMSO treated); carrageenan (0.5 mL of 1.5% (w/v) carrageenan in saline) treated; carrageenan + celecoxib (20 mg/kg body weight) treated; carrageenan + isoorientin (10 mg/Kg body weight) treated; carrageenan + isoorientin (20 mg/Kg body weight) treated.

### 2.9. Histology of Air Pouch Tissue

Air pouch tissues from control and experimental animals were rinsed in PBS and fixed in Bouin's fixative (70% saturated picric acid, 25% formaldehyde, and 5% glacial acetic acid) overnight followed by thorough washing with distilled water. Tissues were then dehydrated sequentially in 70%, 80%, and 90% alcohol and finally in absolute alcohol for 10 min each. After dehydration, the tissue was processed in alcohol and benzene (3 : 1 for 10 min, 1 : 1 for 10 min, benzene and paraffin (1 : 1) for 10 min) to embed in paraffin wax. The tissue was placed in molten paraffin for 2-3 h to allow infiltration of paraffin into the tissue and then allowed to harden. Thin sections (10 *μ*m) were taken on Leitz microtome and mounted on polylysine-coated slides. Sections were deparaffinised by incubating in xylene for 10 minutes and rehydrated by sequential incubations in 90, 80, and 70% alcohol for 10 minutes each. The tissue sections were observed under light microscope at 400x magnification and photographs were taken.

### 2.10. SDS-PAGE and Western Blotting

Pouch tissue homogenate was prepared by homogenizing the pouch lining tissue in 100 mM Tris-HCl (pH 8.0) buffer containing 0.3 M mannitol, 1 mM EGTA, 1 mM EDTA, 4 mM K_2_HPO_4_, 1 mM DTT, 1 mM sodium orthovanadate, 0.1% SDS, 2 mM PMSF, and 40 *μ*L/mL of complete protease inhibitor solution. The homogenate was centrifuged for 30 min at 10,000 rpm at 4°C and the resultant supernatant was used for SDS-PAGE and Western blot analysis. Protein content in the supernatant was measured by Lowry method [16]. SDS-PAGE and Western blot analyses for the detection of COX- 2, iNOS, IL-1*β*, TNF-*α* 5-LOX, and *β*-actin in the air pouch tissue homogenate were performed by the procedure mentioned earlier.

### 2.11. Catalase Assay

Catalase activity was measured using standard protocol described earlier by Aebi [17]. The activity of catalase present in the samples was determined by the decomposition of H_2_O_2_, which can be monitored at 240 nm. 30 mM H_2_O_2_ was mixed in 50 mM phosphate buffer. The assay mixture contained 10 *μ*L of sample and 1 mL of 30 mM H_2_O_2_. The change in the optical density (OD) was monitored for 3 min at 30 sec intervals. The activity of catalase in the diluted sample was calculated using the first-order reaction:

(1) $$\begin{array}{c} K_{30}=\left(\;\frac{2.303}{30}\;\right)*\log\left(\;\frac{A_{1}}{A_{2}}\;\right). \end{array}$$

The activity in the sample was expressed as* K*_30_/mg protein.

### 2.12. Estimation of Glutathione-S-Transferase (GST) Activity

Estimation of GST was carried out using the methodology described earlier by Jakoby [18]. GST catalyses the formation of a conjugate between GSH and a variety of substrates. In this method, 1-chloro-2,4-dinitrobenzene was used as the substrate. The formation of GSH-CDNB catalysed by GST was monitored at 340 nm and the amount of the conjugate formed is a measure of the enzyme activity. The reaction mixture contained 924 *μ*L of phosphate buffer pH, 33.3 *μ*L of CDNB solution (10 mM), and 10 *μ*L of the sample. The reaction was initiated by adding 33.3 *μ*L of GSH solution (10 mM) to the reaction mixture and the change in the OD was monitored at 340 nm for 5 min. The activity of the enzyme in the sample was calculated using the following formula:

(2) GST  activity=Abs  difference ΔD/min×1×100  dilution  factor7.6×5×protein  in  mg.

Activity of GST was expressed as *μ*moles CDNB-GSH conjugate formed/min × mg protein.

## 3. Statistical Analysis

The number of animals used in each treatment group is six (*n* = 6). Data were expressed as mean ± standard error. Correlations between the various parameters were analyzed using regression analysis. *p* value was determined by the Student's *t*-test. *p* value of less than 0.05 was considered as a significant difference.

## 4. Results

### 4.1. Effect of Isoorientin on RAW 264.7 Cell Viability

**Figure 1:**
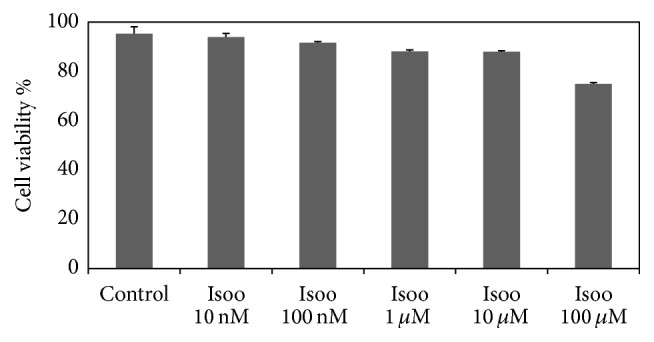
Effect of isoorientin on RAW 264.7 cells viability. The cells were incubated with or without isoorientin along with LPS (1 *μ*g/mL) for 16 hrs and then the cell viability as measured by MTT assay was determined. The percent cell growth was calculated in comparison with untreated control cells. Data are mean ± SEM of three independent experiments (*N* = 3).

To study the cytotoxicity of isoorientin, RAW 264.7 cells were incubated with different concentrations of isoorientin (10 nM to 100 *μ*M) along with LPS (1 *μ*g/mL) for 16 h. No significant effect of isoorientin was observed on the growth of RAW cells, suggesting no cytotoxicity of isoorientin up to 100 *μ*M (Figure 1).

### 4.2. In Vitro Effect of Isoorientin on the Expression of COX-2, iNOS, 5-LOX, TNF-*α*, and IL1-*β*

**Figure 2:**
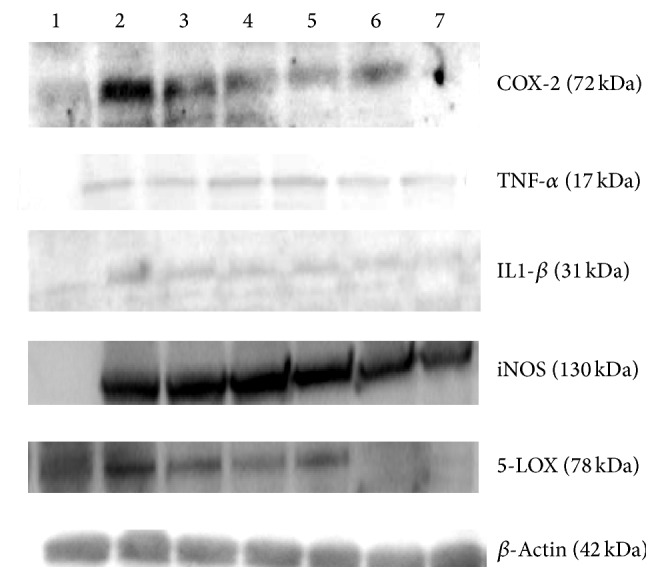
In vitro effect of different isoorientin concentrations on the expression of COX-2, TNF-*α*, IL-1-*β*, iNOS, and 5-LOX, by Western blot. (lane 1) Control cells, (lane 2) LPS alone (lane 3), LPS + celecoxib, (lane 4) LPS + Isoorientin 1 *μ*M, (lane 5) LPS + Isoorientin 5 *μ*M, (lane 6) LPS + Isoorientin 10 *μ*M, and (lane 7) LPS + Isoorientin 25 *μ*M.

Expression levels of inflammatory proteins like COX-2, iNOS, 5-LOX, TNF-*α*, and IL1-*β* were studied in RAW 264.7 cells pretreated with isoorientin and then challenged with or without LPS. RAW 264.7 cells were pretreated with 1 *μ*M, 5 *μ*M, 10 *μ*M, and 25 *μ*M concentrations of isoorientin and after 3-4 h of preincubation cells were induced with 1 *μ*g/mL LPS. Control cells did not receive any LPS. Celecoxib was used as the standard anti-inflammatory drug. The results revealed increased expression of all the inflammatory markers studied in cells treated with LPS. However, the expression levels were decreased in isoorientin treated cells in a dose dependent manner (Figure 2) and isoorientin showed decreased expression of inflammatory proteins better than standard drug.

### 4.3. Effect of Isoorientin on LPS Induced NF-*κ*B Activation

**Figure 3:**
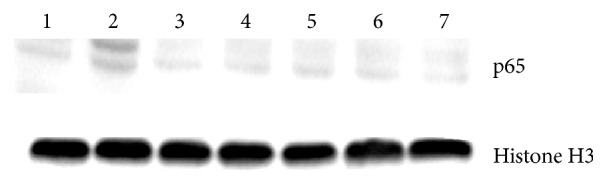
Effect of isoorientin on translocation of NF-*κ*B in LPS stimulated RAW 264.7 cells. Nuclear extracts were prepared from controls or pretreated with different concentrations of isoorientin for 1 h and then induced with LPS for 1 h and analyzed for NF-*κ*B translocation.

As NF-*κ*B is required for the activation of inflammatory proteins like COX-2 and iNOS, Western blot was performed to check whether isoorientin suppresses LPS induced NF-*κ*B activation. Since p65 is one of the major components of NF-*κ*B, we examined the translocation of NF-*κ*B from cytosol to nucleus by Western blotting. Negligible levels of p65 were detected in control nuclei, but treatment with LPS alone for 1 h resulted in marked increase of p65 levels in the nucleus. Pretreatment with isoorientin or celecoxib inhibited LPS induced nuclear translocation of p65 subunit of NF-*κ*B in a concentration dependent manner (Figure 3).

### 4.4. Isoorientin Inhibits Paw Swelling in a Murine Paw Edema Model

**Figure 4:**
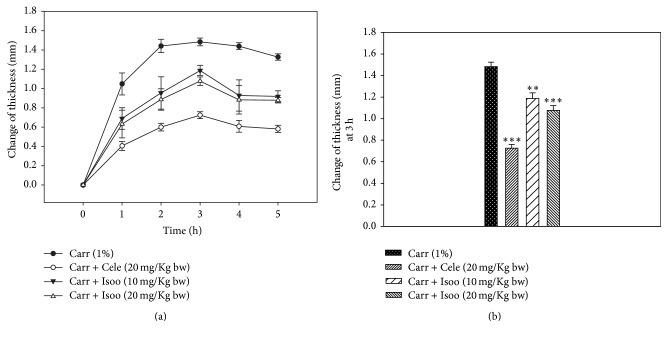
Effect of isoorientin on carrageenan induced paw edema in BALB/c mice. Animals were injected with isoorientin (10 and 20 mg/Kg body weight), celecoxib (20 mg/Kg body weight), or an equal volume of the vehicle (50 *μ*L, 0.2% DMSO) intraperitoneally. One hour later, paw inflammation was induced by injecting 25 *μ*L of 1% solution of carrageenan in 0.9% saline subcutaneously into the plantar region of the left hind paw. (a) The thickness of the paw was measured in the dorsal plantar axis at the metatarsal level by digital calliper at the indicated times after carrageenan injection. (b) The thickness of the paw edema at 3 hour after induction was shown. All data are expressed from *n* = 6 as mean ± SD. ^*∗∗*^*p* < 0.01; ^*∗∗∗*^*p* < 0.001 compared with the control.

Paw edema was induced in mice using carrageenan, and mice were treated with isoorientin. Celecoxib was used as a positive control. Intraplantar injection of carrageenan caused an increase in the thickness of mouse paw. This paw edema peaked at 3 hour after carrageenan induction, with a thickness of 1.48 ± 0.04 mm and 0.73 ± 0.03 mm in celecoxib group. Animals treated with isoorientin at 10 mg/kg and 20 mg/kg body weight had a statistically significant reduction in paw edema, with a mean peak thickness of 1.19 ± 0.05 mm and 1.08 ± 0.04 mm, respectively. This indicated that isoorientin significantly attenuated paw edema compared with the control group (Figures 4(a) and 4(b)).

### 4.5. Isoorientin Inhibits Inflammation in a Murine Air Pouch Model

**Figure 5:**
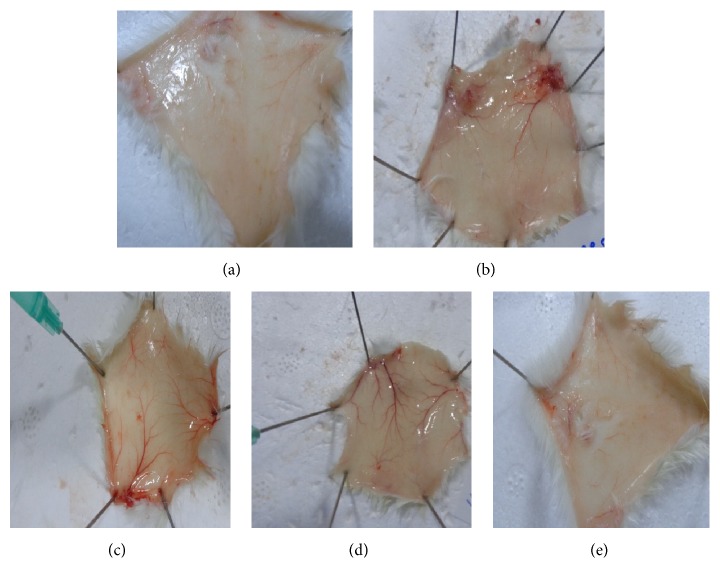
Effect of isoorientin on carrageenan induced blood vessel swelling in air pouch model in Balb/c mice. Photographs of air pouch tissue 24 hours after administration of (a) DMSO, (b) carrageenan, (c) carrageenan + celecoxib, (d) carrageenan + isoorientin 10 mg/kg body weight, and (e) carrageenan + isoorientin 20 mg/kg body weight.

**Figure 6:**
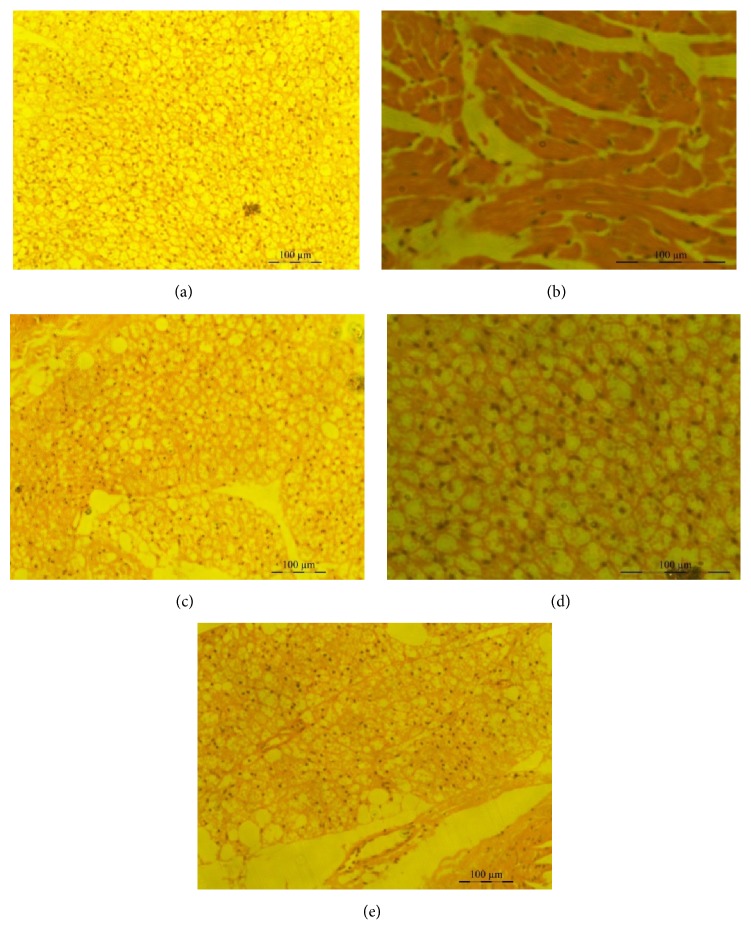
Histopathological observations of granulomatous tissue. Photomicrographs showing the histological sections of pouch tissue 24 hours after administration of (a) DMSO, (b) carrageenan, (c) carrageenan + celecoxib, (d) carrageenan + isoorientin 10 mg/Kg body weight, and (e) carrageenan + isoorientin 20 mg/Kg body weight.

**Figure 7:**
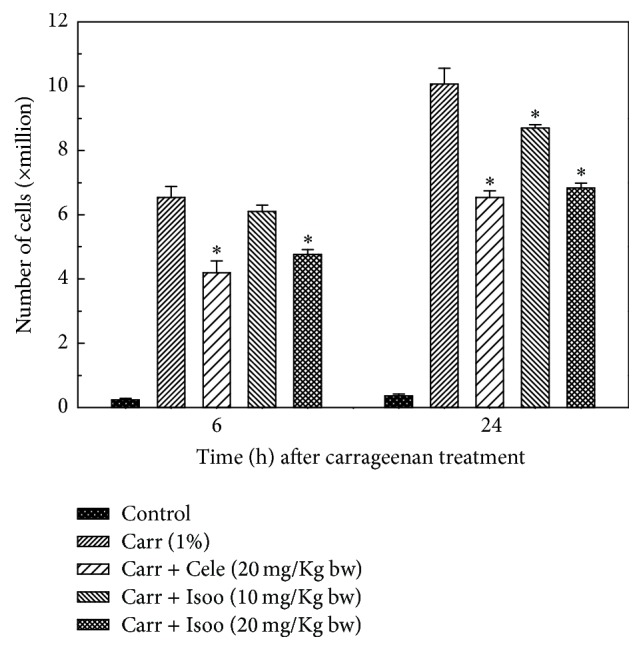
Effect of isoorientin on number of cells infiltrated into the air pouch of either carrageenan or carrageenan + isoorientin treated mice. Animals were sacrificed at various time points after treatments. The values were the mean ± SE of data obtained from 6 different animals. ^*∗*^*p* < 0.05 compared to carrageenan treated animals.

Mice were treated with isoorientin at two different doses of 10 mg/kg body weight and 20 mg/kg body weight along with carrageenan. Control mice received DMSO alone. The classical symptoms of acute inflammation, redness and swelling, were clearly observed in the air pouch lining of carrageenan treated animal. The inflammatory reaction gradually progressed with time and reached a peak at 24 h after carrageenan treatment. In the isoorientin treated animals, the inflammatory reaction was less when compared to animals treated with carrageenan alone. Carrageenan treated pouch tissue showed increases in blood vessel size, while mice tissue of those treated with 10 mg/kg isoorientin showed smaller increases compared to carrageenan treated mice, which was almost completely abrogated in 20 mg/kg isoorientin treated mice (Figures 5 and 6). The number of cells infiltrated in to the air pouch were monitored in carrageenan alone or carrageenan along with isoorientin treated mice. The infiltration of cells increased with carrageenan treatment compared to control. However, this infiltration of cells was decreased when mice were treated with isoorientin in concentration dependent manner (Figure 7). These observations clearly demonstrate the anti-inflammatory effects of isoorientin in the air pouch model of the carrageenan treated animals.

### 4.6. Effect of Isoorientin on the Expression of Inflammatory Proteins

**Figure 8:**
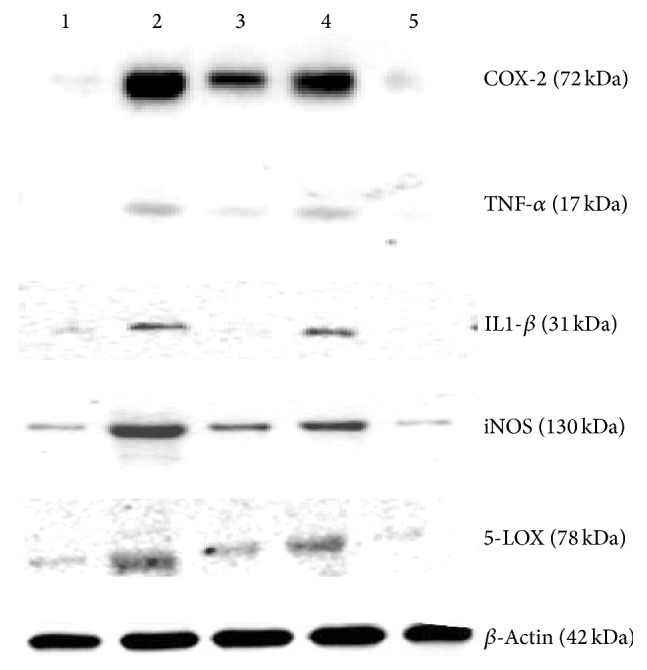
Effect of different isoorientin concentrations on the expression of COX-2, TNF-*α*, IL-1*β*, iNOS, and 5-LOX proteins in the mice air pouch tissue by Western blot. Treatment with (lane 1) DMSO, (lane 2) carrageenan, (lane 3) carrageenan + celecoxib, and (lane 4) carrageenan + isoorientin 10 mg/kg body weight and (lane 5) 20 mg/kg body weight.

As isoorientin reduced inflammation in the mice air pouch model, further we examined the expression of inflammatory proteins COX-2, TNF-*α*, IL-1*β*, iNOS, and 5-LOX by Western blot (Figure 8). The expression of COX-2, TNF-*α*, IL-1*β*, iNOS, and 5-LOX was markedly upregulated in response to carrageenan; however, treatment of isoorientin decreased the expression of these proteins in a concentration dependent manner, and these effects are comparable to those observed with celecoxib treatment.

### 4.7. Effects of Isoorientin on Catalase and Glutathione S-Transferase

**Figure 9:**
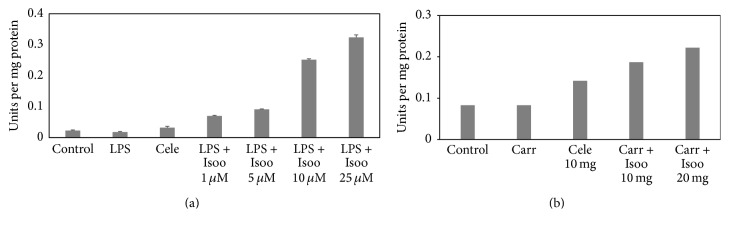
Effect of isoorientin on catalase activity in RAW 264.7 cells and air pouch tissue. Bar graph showing catalase activity in (a) RAW 264.7 cells, (bar 1) control cells, (bar 2) LPS alone, (bar 3) LPS + celecoxib, (bar 4) LPS + isoorientin 1 *μ*M, (bar 5) LPS + isoorientin 5 *μ*M, (bar 6) LPS + isoorientin 10 *μ*M, and (bar 7) LPS + isoorientin 25 *μ*M. (b) Air pouch tissue, (bar 1) control, (bar 2) carrageenan, (bar 3) carrageenan + celecoxib, (bar 4) carrageenan + isoorientin 10 mg/Kg body weight, and (bar 5) carrageenan + isoorientin 20 mg/Kg body weight.

Catalase is critical in catalysing the decomposition of hydrogen peroxide to oxygen and water, thus protecting from oxidative damage due to reactive oxygen species (ROS). The effect of isoorientin on catalase activity was monitored in RAW 264.7 cells as well as tissues from the air pouch of carrageenan treated animals. The results indicate that, in RAW cells, catalase activity showed no significant effects with LPS treatment. However, the catalase activity increased in dose dependent manner in isoorientin treated cells (Figure 9(a)). In the tissues from the air pouch of carrageenan treated animals the catalase activity was similar as observed in control animals, whereas in the tissues of isoorientin treated mice catalase activity increased significantly (Figure 9(b)).

**Figure 10:**
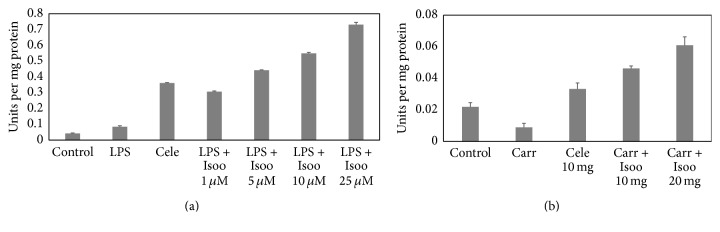
Effect of isoorientin on GST activity in RAW 264.7 cells and air pouch tissue. (a) RAW 264.7 cells, (bar 1) control cells, (bar 2) LPS alone, (bar 3) LPS + celecoxib, (bar 4) LPS + isoorientin 1 *μ*M, (bar 5) LPS + isoorientin 5 *μ*M, (bar 6) LPS + isoorientin 10 *μ*M, and (bar 7) LPS + isoorientin 25 *μ*M. (b) Air pouch tissue, (bar 1) control, (bar 2) carrageenan, (bar 3) carrageenan + celecoxib, (bar 4) carrageenan + isoorientin 10 mg/kg body weight, and (bar 5) carrageenan + isoorientin 20 mg/kg body weight.

GSTs are a group of multigene multifunctional proteins involved in the detoxification of xenobiotics, including organic peroxides. GST activity was increased significantly in a dose dependent manner with isoorientin (Figure 10) in both LPS treated RAW cells and carrageenan induced inflammatory tissue.

## 5. Discussion

Natural products have been used as remedies since ancient times to combat several human disorders such as cardiovascular disease, cancer, and many inflammatory disorders [3, 5]. Drugs derived from natural products are making enormous contribution to drug discovery. Isoorientin is one such natural compound isolated from the tubers of* P. tuberosa*, which is a climbing, coiling, and trailing vine with tuberous roots. Tubers are used to reduce body dryness and for easy bowel movement. Isoorientin was isolated from methanolic extract of* P. tuberosa*. Earlier we have reported that isoorientin inhibited COX-2 with an IC_50 _value of 39 *μ*M [13]. In this study, we have evaluated the anti-inflammatory effects of isoorientin in vitro on mouse macrophage cell line, RAW 264.7, and in vivo on paw edema and air pouch models of inflammation. Although inflammation is a normal response, when it occurs in an uncontrolled or inappropriate manner, excessive damage to host tissues and disease can ensue.

In the present study we showed that isoorientin does not affect the growth of RAW cells up to 100 *μ*M conc. COX-2, iNOS, and 5-LOX serve as key mediators of inflammation. The agents that inhibit the expression of these proteins have therapeutic potential for inflammatory diseases. The expression levels of these proteins were increased on induction with LPS and were decreased on isoorientin treatment in dose dependent manner. TNF-*α* and IL-1*β* are known to have key role in inflammatory processes and are mainly produced by macrophages [19]. In the present study isoorientin treatment significantly reduces the expression of TNF-*α* and IL-1*β* in a concentration dependent manner. NF-*κβ* is known to control the expression of cell survival genes, cytokines and proinflammatory markers [19]. Isoorientin inhibited the LPS induced translocation of NF-*κβ*/p65 as evidenced by Western blotting.

In the air pouch model the inflammatory reaction gradually progressed with time and reached a peak at 24 h after carrageenan treatment. Mice treated with isoorientin showed less inflammation compared to carrageenan treated mice. Histopathological studies clearly demonstrate that carrageenan treated pouch tissue showed heavy infiltration of blood cells at various sites in the tissue, whereas isoorientin treated animals, however, showed reduced inflammatory reaction as indicated by less degree of cellular infiltration. Carrageenan treatment increased the expression of COX-2, TNF-*α*, IL-1*β*, iNOS, and 5-LOX and this was decreased by isoorientin treatment in dose dependent manner.

Free radicals can be defined as molecules or molecular fragments containing one or more unpaired electrons in atomic or molecular orbitals [20]. Reactive oxygen species (ROS) include all those reactive radical and nonradical oxygen species which are highly reactive entities and can readily participate in a variety of chemical/biochemical reactions. ROS can be formed in the heart and other tissues, by several mechanisms; they can be produced by xanthine oxidase (XO), NAD(P)H oxidase, cytochrome P450; by autooxidation of catecholamine; and by uncoupling of NO synthase (NOS) [21–24]. There are several cellular mechanisms that counterbalance the production of ROS, including enzymatic and nonenzymatic pathways [25]. Among the best-characterized enzymatic pathways are catalase and glutathione-S-transferase (GST). Nonenzymatic mechanisms include intracellular antioxidants such as the vitamins E, C, and *β*-carotene (a precursor to vitamin A), ubiquinone, lipoic acid, and urate [25]. We found that isoorientin is able to increase catalase activity in tissues of isoorientin treated mice. We also found that isoorientin increased GST activity that was decreased on carrageenan treatment.

## 6. Conclusions

Isoorientin, selective COX-2 inhibitor isolated from tubers of* P. tuberosa*, showed potent anti-inflammatory properties in vitro on mouse macrophage cell line, RAW 264.7, challenged with LPS. Also isoorientin was found to be effective in reducing the carrageenan induced inflammation in vivo on paw edema as well as air pouch mouse models. These effects of isoorientin appear to be mediated by the inactivation of NF-*κ*B and downregulating the expression of proinflammatory genes such as COX-2, iNOS, and TNF-*α* and activation of antioxidant defense enzymes such as catalase and GSTs.

## Abbreviations

COX: Cyclooxygenase
COXIBS: Cyclooxygenase inhibitors
DMSO: Dimethyl sulfoxide
GST: Glutathione-S-transferase
IL: Interleukin
iNOS: Inducible nitric oxide synthase
LPS: Lipopolysaccharide
MTT: 3-(4,5-Dimethylthiazol-2-yl)-2,5-diphenyltetrazolium bromide
NF-*κ*B: Nuclear factor kappa B
NSAIDs: Nonsteroidal anti-inflammatory drugs
TNF-*α*: Tumor necrosis factor-*α*.
